# Histone deacetylase inhibitor, panobinostat, exerts anti-proliferative effect with partial normalization from aberrant epigenetic states on granulosa cell tumor cell lines

**DOI:** 10.1371/journal.pone.0271245

**Published:** 2022-07-08

**Authors:** Yukiko Hazama, Takayuki Tsujioka, Akira Kitanaka, Kaoru Tohyama, Koichiro Shimoya

**Affiliations:** 1 Departments of Obstetrics and Gynecology, Kawasaki Medical School, Okayama, Japan; 2 Department of Laboratory Medicine, Kawasaki Medical School, Okayama, Japan; Saint Louis University School of Medicine, UNITED STATES

## Abstract

The prognosis of the patients with inoperable or advanced granulosa cell tumors (GCTs) is still poor, and therefore it is important to establish a novel treatment strategy. Here we investigated the *in vitro* effects of a histone deacetylase inhibitor, panobinostat (PS) on two GCT cell lines (KGN and COV434). GCT cell lines were found to be susceptible to PS treatment and it inhibited cell growth mainly by apoptosis. In cell cycle analysis, PS reduced only the ratio of S phase in GCT cell lines. Combined treatment of PS with a deubiquitinase inhibitor, VLX1570 enhanced the expression of p21, cleaved PARP, cleaved caspase-9, heme oxygenase-1, and the acetylation of histone H4 and α-tubulin, leading to an additive anti-proliferative effect on KGN and COV434. The gene set enrichment analysis revealed that PS treatment suppressed DNA replication- or cell cycle-related gene expression which led to chemotherapeutic cell death and in addition, this treatment induced activation of the gene set of adherens junction towards a normalized direction as well as activation of neuron-related gene sets that might imply unexpected differentiation potential due to epigenetic modification by a HDAC inhibitor in KGN cells. Exposure of KGN and COV434 cells to PS increased the expression of E-cadherin, one of the principal regulators associated with adherens junction in quantitative RT-PCR and immunoblotting analysis. In the present study, we indicate a basis of a novel therapeutic availability of a HDAC inhibitor for the treatment of GCTs and further investigations will be warranted.

## Introduction

Granulosa cell tumors (GCTs) account for approximately 70% of malignant sex cord–stromal tumors and represent 3 to 5% of all ovarian neoplasms. GCTs frequently occur in perimenopausal women and a half of those show atypical genital bleeding and menstruation disorder from the overproduction of estrogen [1, 2]. A somatic missense mutation (C134W) of *FOXL2* which is involved in follicular development and granulosa cell differentiation is found in 95% of adult patients with GCTs [3]. Since more than 80–90% of the patients with GCTs is diagnosed at an early phase and can achieve remission surgically by removing the localized lesion and the five-year survival rate is more than 95% at stages I and II, while it worsens to 59% at stages III and VI. Although BEP therapy (bleomycin, etoposide and cisplatin) as a representative adjuvant chemotherapy is performed after surgery at stage II-VI, the response rate of this treatment is barely 37% [4]. Therefore, it has been expected to establish a novel chemotherapeutic strategy for the patients with advanced, recurrent or metastatic GCTs.

The aberrant gene expression by epigenetic change plays a critical role in the onset and progression of cancer [5]. Histone acetyltransferases (HATs) transfer acetyl groups to amino-terminal lysine residues in histone, which results in increased transcriptional activity, whereas histone deacetylases (HDACs) catalyze the removal of acetyl groups, leading to transcriptional repression [6, 7]. HDACs has been recognized as targets to reverse aberrant epigenetic states that are involved in oncogenesis [8].

Panobinostat (PS) is a HDAC inhibitor classified into hydroxamate and strongly inhibits enzymatic activity of HDACs belonging to class I, II and IV [9]. It was previously reported to show an anti-proliferative effect on acute myeloid leukemia, Hodgkin’s disease, multiple myeloma (MM) and various solid tumors [10–13]. Furthermore, it was reported that the combined treatment of PS with conventional therapeutic agents including gemcitabine or paclitaxel showed a synergistic effect on ovarian cancer cell lines [14–16].

In the present study, we investigated an anti-proliferative effect of PS on GCT cell lines and indicated that PS induced apoptosis and the combined treatment with a deubiquitinase inhibitor, VLX1570 showed an additive cytoreductive effect. The gene set enrichment analysis (GSEA) revealed that PS treatment suppressed DNA replication- or cell cycle-related gene expression which led to chemotherapeutic cell death and further induced activation of the gene set of adherens junction towards a normalized direction as well as activation of neuron-related gene sets that might imply unexpected differentiation potential due to epigenetic modification by a HDAC inhibitor in KGN cells.

## Materials and methods

### Reagents

PS and VLX1570 were purchased from Selleck Chemicals, (Houston, Texas, TX, USA). This was dissolved in dimethylsulfoxide and stored at -80°C with being protected from light. We used those at the concentrations up to 200 nM.

### Cell lines and culture

Granulosa cell tumor cell lines, KGN and COV434 were maintained in DMEM/Ham’s F12 medium supplemented with 10% fetal bovine serum. An ovarian serous adenocarcinoma cell line, SK-OV-3 and a clear cell cancer cell line, RMG-I and OVISE were used in this study. Six human normal bone marrow CD34-positive progenitor cells were purchased from LONZA Group Ltd, Basel, Switzerland and cultured with RPMI1640 medium supplemented with 10% fetal bovine serum and with recombinant cytokines for myelopoiesis of hematopoietic progenitor cells.

### Cell growth assay and MTT assay

Cell count was assessed by MTT assay. Cell suspensions were plated into 96-well plates in the presence of the drug or solvent alone, incubated as above at 37°C for 1–4 days, and analyzed by the 3-(4,5-dimethythiazol-2-yl)-2,5-diphenyl tetrazolium bromide (MTT) assay [17]. Quintuplicate experiments by the technical replicates were performed.

### Apoptosis assay

Apoptosis was examined using an AnnexinV Apoptosis Detection Kit (BD Pharmingen, San Diego, CA, USA) and all samples were analyzed with FACS Calibur flowcytometer and CellQuest software (Becton Dickinson, Franklin Lakes, NJ, USA) [18]. Triplicate experiments by the biological replicates were performed.

### Cell cycle analysis

Cells were fixed with 70% methanol for 30 min and treated with 2 mg/ml ribonuclease A (Nacalai Tesque, Kyoto, Japan) for 30 min at 37°C, then with 50 μg/mL propidium iodide (PI; Sigma, St Louis, MO, USA) for further 20 min at room temperature [19]. Triplicate experiments by the biological replicates were performed.

### Immunoblotting analysis

Cell lysates of all five cell lines were prepared in lysis buffer containing 50 mM Tris-HCL, 150 mM NaCl, 5 mM EDTA, 0.5% TritonX-100, 0.05% sodium dodecyl sulfate (SDS), 0.5% sodium deoxycholate, 2 mM phenylmethylsulfonyl fluoride and 1 mM Na_3_VO_4_. The lysates were separated by SDS-polyacrylamide gel electrophoresis (SDS-PAGE) and immunoblotting analysis was performed as previously described [20].

Membranes were incubated with a 1:1000 dilution of primary antibodies in Can Get Signal solution 1 (TOYOBO, Osaka, Japan), followed by corresponding secondary antibodies (1:1000 dilution with Can Get Signal solution 2). Primary antibodies were obtained from Cell Signaling Technology (cleaved-PARP (cPARP), p21, acetyl-H4 (Lys12), E-cadherin; Danvers, MA, USA), Sigma-Aldrich (α-tubulin, acetyl-α-tubulin (Lys40); St. Louis, Mo, USA), Horse-radish peroxidase-conjugated mouse and rabbit antibodies were from GE Healthcare Life Sciences (Piscataway, NJ).

### Gene expression profiling and gene set enrichment analysis (GSEA)

Gene expression profiling of KGN cells were analyzed in three independent experiments. Treated cells were harvested after 24 h treatment with 100 nM of PS. Total RNA was extracted with RNeasy Mini Kit (Qiagen, Germantown, MD, USA) and RNase-Free DNase Set (QUIAGEN, Hilden, Germany) in on-column digestion of DNA, converted to cDNA and amplified with GeneChip WT Terminal Labeling and Controls Kit (Affymetrix, Santa Clara, CA, USA). The fragmentation, the labeling and the hybridization of cDNA were treated with GeneChipTM Human Gene 2.0 ST Array (Thermo Fisher Scientific, Inc.). Chips were scanned with a GeneChip Scanner 3000 7G System (Affymetrix). We submitted this gene expression profiling to Gene Expression Omnibus. Its accession number is GSE201203.

The gene set enrichment analysis (GSEA; Broad Institute Cambridge, MA, USA) was performed using the gene expression profiling data and by handling the GSEA software (GSEA software v4.1.0 for Windows). The detail information of these experiments is described in references [21]. In this study, the whole expression change in the gene sets was defined as statistically significant if the false discovery rate (FDR) q-value was less than 0.25.

### Quantitative real-time reverse transcription PCR

Quantitative real-time reverse transcription PCR (q-PCR) was performed with Applied Biosystems StepOne PlusTM Real time PCR system (Life Technologies Japan Ltd, Tokyo). Total cellular RNA (1–2 μg) was used to synthesize cDNA by using Ready-To-Go T primed First-Strand Kit (GE Healthcare UK Ltd, Amersham Place, Little Chalfont, Buckinghamshire HP7 9NA, UK) in a final volume of 33 μl. Five μl of 1:10 diluted cDNA reactions was used as input for each of the real-time quantitative PCR reactions by using SYBR Premix Ex TaqTM (Takara Bio Inc, Shiga, Japan). Initial denaturation at 95°C for 20 s was followed by 40 cycles of a denaturation step at 95°C for 3 s and an annealing and extension step at 60°C for 30 s [22].

*CDH1*, official symbol of *E-cadherin* (NM_ 004360.5) forward primer was 5’-gaagtgactcgtaacgacgttg-3’ and its reverse primer was 5’- cacgagcagagaatcataaggc-3’. Human housekeeping gene *RPL27* (NM_000988) was used as endogenous control. Primers for *RPL27* were as follows: forward: 5’-CTCTGGTGGCTGGAATTGAC-3’ and reverse: 5’-AAACCGCAGTTTCTGGAAGA-3’. Triplicate experiments by the technical replicates were performed.

### Statistical analyses

All results are shown as the mean values with ranges. Comparisons between the groups were done using the Dunnett’s and Scheffe’s tests. Differences were considered statistically significant if p-values were less than 0.05. These analyses were carried out using SPSS for Windows version 14.0.

## Results

### PS inhibits the proliferation of ovarian cancer and GCT cell lines and mainly induces apoptosis

**Fig 1:**
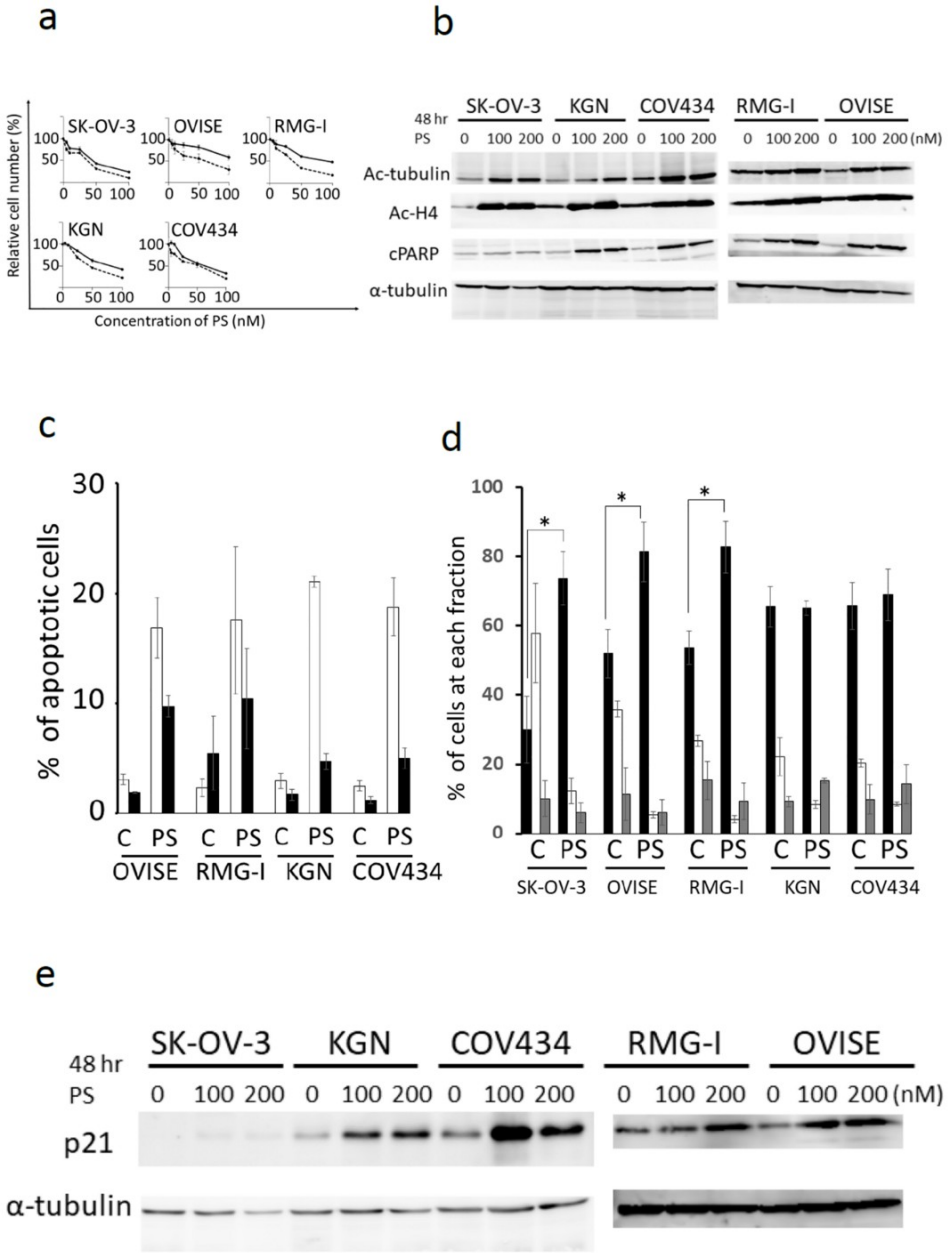
PS inhibits the proliferation of GCT and ovarian cancer cell lines and induces apoptosis. a) Three ovarian cancer cell lines (SK-OV-3, OVISE and RMG-I) and two GCT cell lines (KGN and COV434) were cultured with PS (0-100nM) for indicated times (48 and 72 hr). Cell viability was estimated by MTT assay. The value without PS was adjusted to 100%. The data represent the mean values with SD from five independent experiments. solid line:48hr, dash line:72hr. b) The cell lysates of each cell line were analyzed by immunoblotting analysis for detection of acetyl-α-tubulin, acetyl-histone H4 and cleaved PARP. α-tubulin was used as a loading control. Ac-tubulin (acetyl-α-tubulin), Ac-H4 (acetyl-histone H4), cPARP (cleaved PARP). c) Four cell lines were cultured with or without 100nM of PS for 48 hr and apoptosis was assessed by flow cytometry using annexin V/PI dual staining. The single-positive fraction for annexin V (white bar) implies early apoptosis, and the double-positive fraction for annexin V/PI (black bar) implies late apoptosis. The data represent the mean values with SD from three independent experiments. C: DMSO control, PS: 100 nM of PS. d) The cell cycle analysis by flow cytometory is shown. Five cell lines were treated with 100 nM of PS for 24 h, and cells were stained with PI and analyzed by flow cytometry. The data represent the mean values with SD from three independent experiments. The cell fractions at G0G1, S and G2/M phase are presented by black, white and gray bars, respectively. C: DMSO control, PS: 100 nM of PS. e) The cell lysates of each cell line were analyzed by immunoblotting analysis for detection of p21. α-tubulin was used as a loading control. In d), statistical differences were evaluated and presented by asterisks in some cell lines, if *p*-values were less than 0.05.

To determine whether PS inhibits the proliferation of ovarian cancer and GCT cells, we cultured three ovarian cancer cell lines including SK-OV-3, OVISE and RMG-I and two GCT cell lines including KGN and COV434 with 0–100 nM of PS for 0–72 hr and examined the halfmaximal inhibitory concentration (IC50) of PS in all 5 cell lines (48 and 72hr) with MTT assay (Fig 1a). The IC50 value of PS for SK-OV-3, OVISE, RMG-I, KGN and COV434 at 72 h was 34.4±0.11, 44.0± 0.46, 58.5±1.0, 34.7±0.94 and 53.5±8.4 nM, respectively (Table 1). We found that all 5 cell lines were susceptible to PS (Table 1). To evaluate the effect of PS on normal cells, we treated six human normal bone marrow CD34-positive progenitor cells with 0–100 nM of PS for 0–96 hr and confirmed that 50–100 nM of PS inhibited the proliferation of CD34+ progenitor cells by cell counting using trypan blue staining (S1 Fig). Since PS affects normal cells under the same concentration for cancer cells, we recommend to use this agent at low dose. PS also induced the acetylation of Histone H4 and α-tubulin in SK-OV-3, RMG-I, OVISE, KGN and COV434 cell lines (Fig 1b and S2 Fig).

**Table 1:** IC50 (nM) of PS in GCT and ovarian cancer cell lines.

	48 hr	72 hr
SK-OV-3	43.1±1.3	34.4±0.11
OVISE	*	58.5±1.0
RMG-I	75.2±1.8	44.0±0.46
KGN	89.5±1.0	34.7±0.94
COV434	59.4±1.4	53±8.4

PS: Panabinostat, GCT* Granulosa Cell Tumors.

*Implies that exposure of OVISE to PS did not reach the IC_50_ in spite of being treates by the maximal concentration (100nM)

Next, we examined the degree of apoptosis of cells treated with PS by dual staining of annexinV and PI and confirmed that it induced apoptosis in 4 cell lines (Fig 1c). The amount of cleaved PARP (cPARP), a marker of cells undergoing apoptosis, was increased with 100 or 200nM of PS treatment at 48 hr in SK-OV-3, RMG-I, OVISE, KGN and COV434 cell lines (Fig 1b).

Since PS has been previously reported to affect the cell cycle [10–13], we performed the cell cycle analysis of PS-treated cell lines by flow cytometry and demonstrated that exposure of ovarian cancer cells including SK-OV3, OVISE and RMG-I to PS for 24 h led to the increase in the cell fraction at G0/G1 phase, whereas exposure of GCT cell lines including KGN and COV434 to PS did not significantly increase the G0/G1 cell fraction in spite of obvious decrease of cell fraction at S phase (Fig 1d). In immunoblotting analysis, Exposure of SK-OV-3, RMG-I, OVISE, KGN and COV434 to 0-200nM of PS for 48 hr increased the expression of cyclin dependent kinase inhibitor, p21 (Fig 1e and S3 Fig).

### Deubiqutinase inhibitor, VLX1570 and PS trigger additive cytotoxicity in GCT cell lines

**Fig 2:**
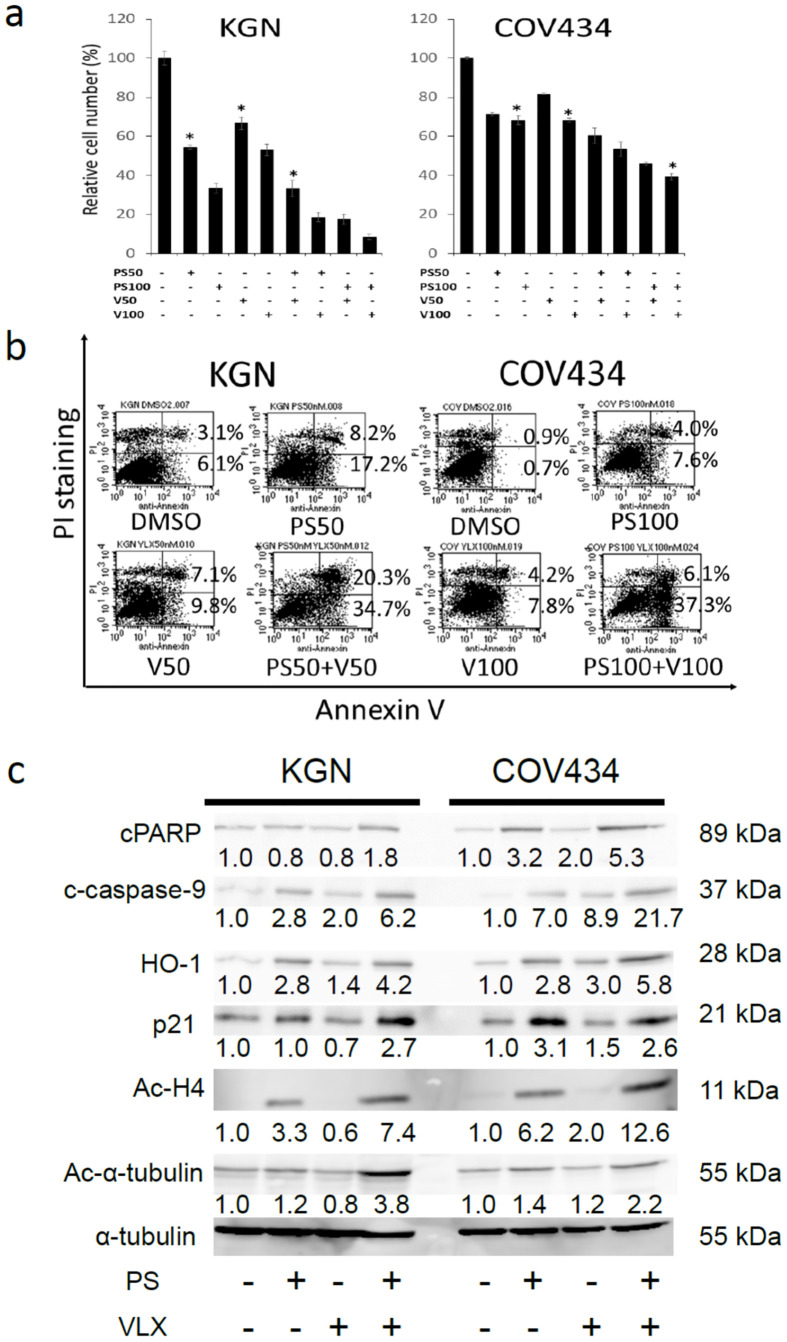
The combined treatment of PS with a deubiqutinase inhibitor, VLX1570 showed the additive effect on KGN and COV434 cell lines. a) KGN and COV434 were cultured with PS (0–100 nM), VLX1570 (0–100 nM) or both for 48 hr. Cytotoxicity was estimated by MTT assay. The data represent the mean values with SD from five independent experiments. Statistical differences were evaluated and presented by asterisks in two cell lines, if *p*-values were less than 0.05. PS: panobinostat, V: VLX1570. b) KGN and COV434 were cultured with PS (0–100 nM), VLX1570 (0–100 nM) or both for 48 hr. The fraction of apoptosis cells was measured by flow cytometry analysis using annexin V/ PI dual staining. PS: panobinostat, V: VLX1570. c) KGN and COV434 were cultured with PS (0–100 nM), VLX1570 (0–100 nM) or both for 24 hr. The cell lysates of each cell line were analyzed by immunoblotting analysis for detection of p21, cleaved PARP (cPARP), cleaved caspase-9 (c-caspase-9), heme oxygenase-1 (HO-1), acetylated histone H4 (Ac-H4) and acetylated α-tubulin (Ac-α-tubulin). α-tubulin was used as a loading control. The number of protein bands was measured by densitometry and the ratio was adjusted as 1.0 in the untreated sample and the changes of the ratio of treated samples relative to untreated sample were indicated.

The ubiquitin proteasome system (UPS) is the procedure which degrades cellular unfolded or misfolded proteins distinct from autophagy lysosome system [23, 24]. The inhibition of UPS by deubiqutinase inhibitor, b-AP15 or VLX1570 has been previously reported to induce apoptosis in various cancer cells [25–29]. We cultured KGN and COV434 cells with the combined treatment of PS with VLX1570 and demonstrated that VLX1570 enhanced the growth-suppressive effect of PS on MTT assay (Fig 2a). This combined treatment additively increased the number of apoptotic cells in flow cytometry analysis using dual staining of annexinV and PI (Fig 2b). To further determine the mechanism of additive effect by the combined treatment of PS with VLX1570, we examined proteins related to cell cycle progression, apoptosis, the generation of ROS and the histone acetylation by immunoblotting analysis. The combined treatment enhanced the expression of cPARP, cleaved caspase-9, heme oxygenase-1 (HO-1), and the acetylation of histone H4 and α-tubulin in KGN and COV434 (Fig 2c and S4 Fig), whereas it enhanced the expression of p21 in KGN but not in COV434. 100 nM of PS alone might have caused strong p21 expression in COV434.

### PS possibly restores the suppressed expression of the gene sets associated with adherens junction and neurotransmitter release cycle

To further explore the action mechanisms of PS, we examined the gene expression profiling of KGN cells treated with or without 100 nM of PS for 24 hr. Genes whose expression changed by more than twofold Fold (Change (FC) >2.0, FC<0.5, p<0.05) following drug treatment were defined as regulated genes. 1630 genes were upregulated, and 1590 genes were downregulated in KGN cells treated with 100 nM of PS compared with the control.

**Fig 3:**
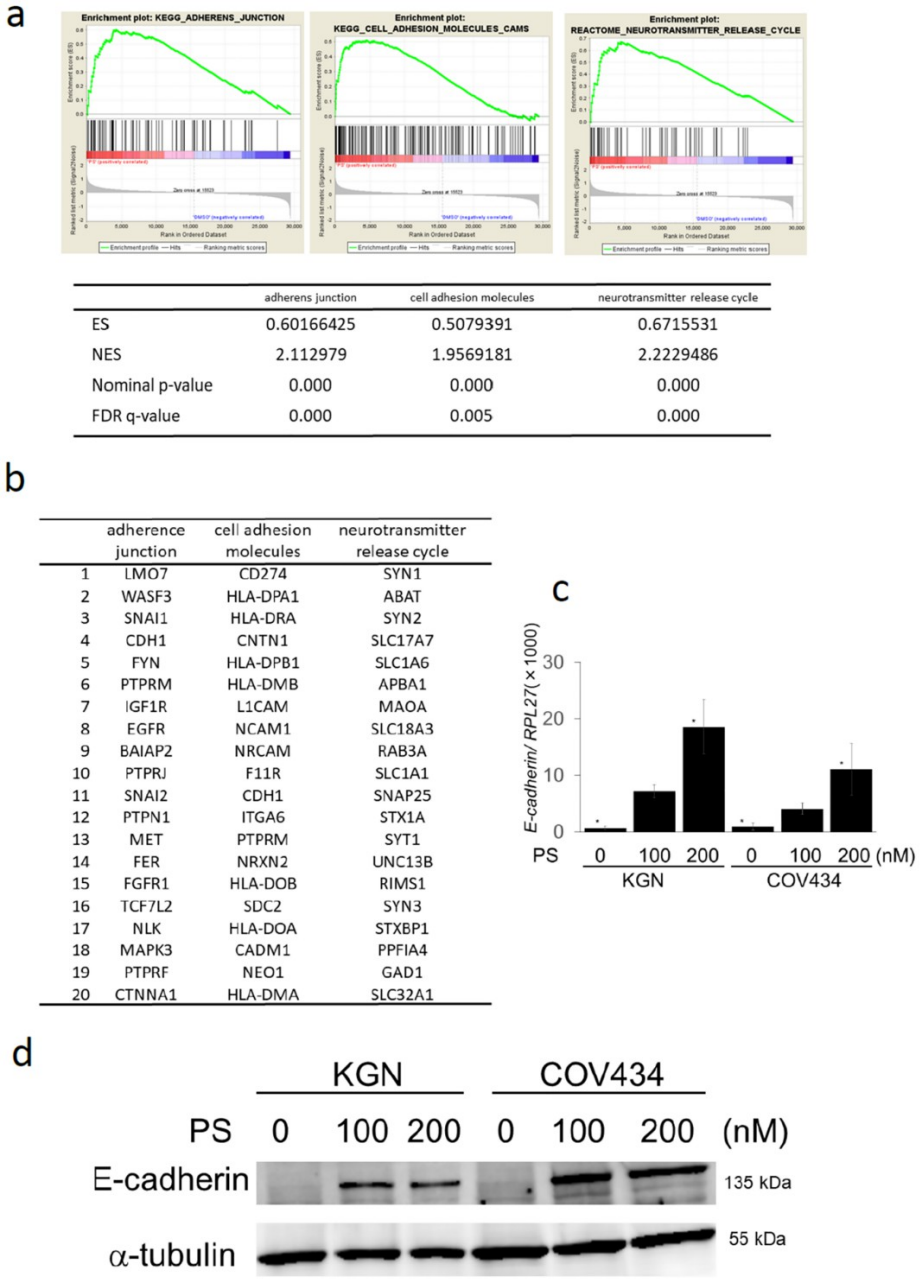
The gene enrichment analysis (GSEA) revealed the up-regulated gene sets: “Adherens junction”, “cell adhesion molecules”, and “neurotransmitter release cycle” in PS-treated KGN cells. a) PS (100 nM)-treated or untreated KGN cells were harvested at 24 hr. Gene expression profiling of KGN cells was examined in triplicate experiments and obtained data were used for GSEA by handling the GSEA software and the Molecular Signatures Database according to the references. The gene set “adherens junction”, “cell adhesion molecules” and “neurotransmitter release cycle” was strongly up-regulated by PS treatment and several statistical values are also presented. ES: enrichment score, NES: normalized enrichment score. b) The top 20 of most significant differentially expressed genes detected by GSEA analysis was shown. c) The expression of *E-cadherin* (official symbol: *CDH1*) in KGN and COV434 treated with 0–200 nM of PS for 24 h was examined by quantitative RT-PCR. The copy ratio of *E-cadherin* to *RPL27* was calculated and indicated as a quantification of *E-cadherin* expression. d) The expression of E-cadherin in KGN and COV434 treated with 0–200 nM of PS for 24 h was examined by immunoblotting analysis.

To know the gene targets of PS, we performed GSEA and found that some of the most upregulated GSEA sets were “adherens junction” (FDR q-val 0.000), “cell adhesion molecules” (FDR q-val 0.005) in KEGG pathway and “neurotransmitter release cycle” (FDR q-val 0.000) in reactome pathway (Fig 3a). The top 20 of most significant differentially expressed genes detected by GSEA analysis was shown in Fig 3b. All genes included in each gene set were shown in S5 Fig. Taken together, the gene expression profiling suggested that PS restored the genetic pathways involved in adherens junction, cell adhesion molecules and neurotransmitter release cycle which had been suppressed in KGN cells. Among the genes belonging to “adherens junction” that were found to be upregulated by PS treatment, we focused on E-cadherin (official symbol: *CDH1*) and actually examined its expression by quantitative RT-PCR and immunoblotting analysis. Exposure of KGN and COV434 cells to 100–200 nM of PS for 24 h significantly increased the expression of E-cadherin (p<0.05) (Fig 3c and 3d, S6–S8 Figs).

### PS possibly suppressed expression of the gene sets associated with DNA replication, cell cycle and splicesome

**Fig 4:**
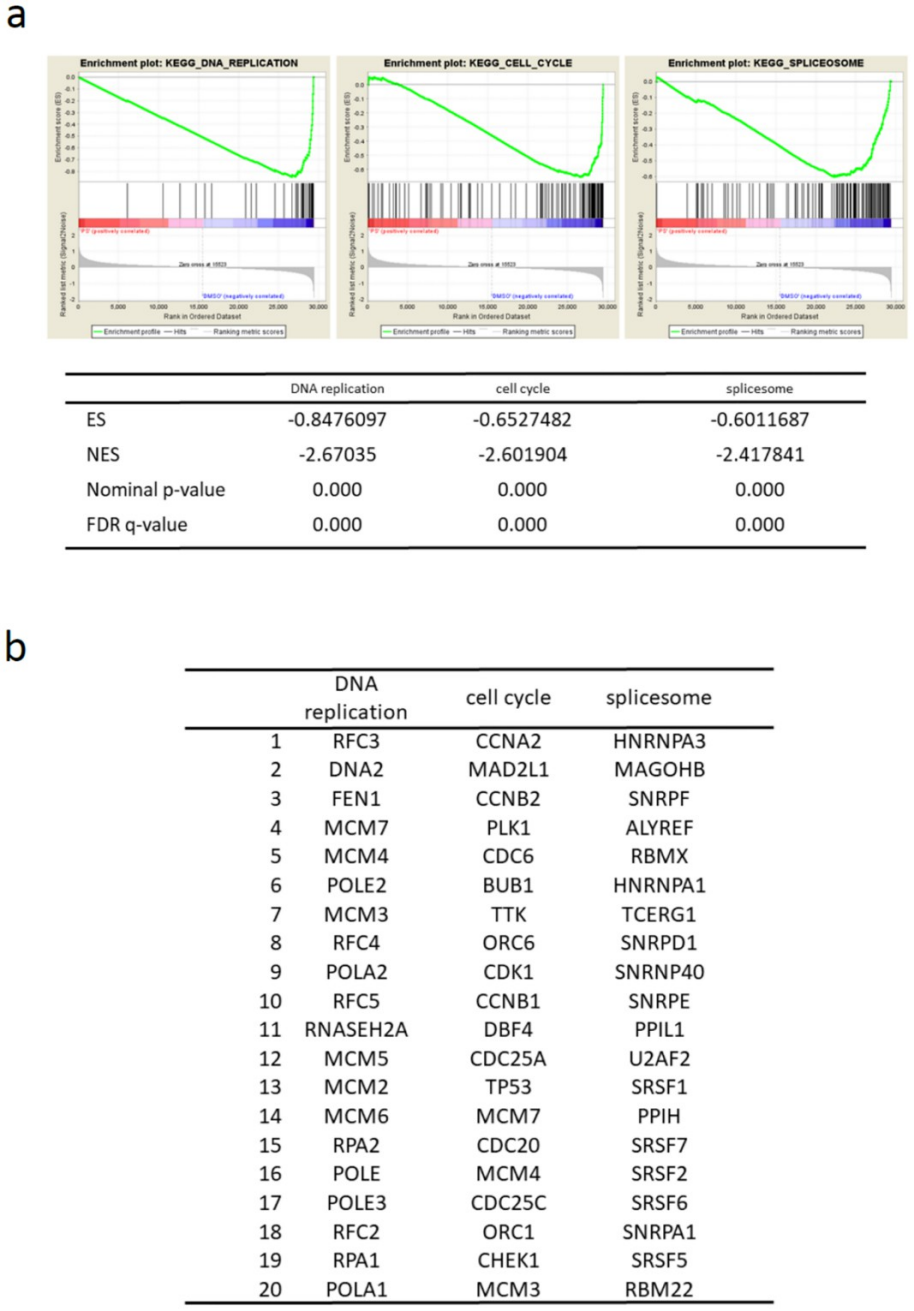
The gene enrichment analysis (GSEA) revealed the down-regulated gene sets: “DNA replication”, “cell cycle”, and “splicesome” in PS-treated KGN cells. a) PS (100 nM)-treated or untreated KGN cells were harvested at 24 hr. Gene expression profiling of KGN cells was examined in triplicate experiments and obtained data were used for GSEA by handling the GSEA software and the Molecular Signatures Database according to the references. The gene set “DNA replication”, “cell cycle”, and “splicesome” was strongly down-regulated by PS treatment and several statistical values are also presented. ES: enrichment score, NES: normalized enrichment score. b) The top 20 of most significant differentially expressed genes detected by GSEA analysis was shown.

We also found that some of the most downregulated GSEA sets were “DNA replication” (FDR q-val 0.000), “cell cycle” (FDR q-val 0.000) and “splicesome” (FDR q-val 0.000) in KEGG pathway (Fig 4a). The top 20 of most significant differentially expressed genes detected by GSEA analysis was shown in Fig 4b. All genes included in each gene set were shown in S9 Fig. Taken together, the gene expression profiling suggested that PS suppressed the genetic pathways involved in DNA replication, cell cycle and splicesome in KGN cells.

## Discussion

Catley et al. investigated the effect of the combined treatment of PS with a proteasome inhibitor, bortezomib on multiple myeloma cells [30]. UPS works for quality control of the proteins by degrading unnecessary misfolded or unfolded proteins. If the amount of unnecessary proteins exceeds the capacity of UPS, they accumulate in aggresomes which are inclusion bodies formed by transport of aggregated protein on microtubules [31, 32]. After HDAC6 binds both ubiquitinated misfolded proteins and motor protein dynein, it carries misfolded proteins to aggresomes along microtubules using dynein [33, 34]. Since inhibition of UPS by bortezomib on myeloma cells leads to aggresome formation and PS inhibits the activity of HDAC6, the combined treatment shows the synergistic effect on myeloma cells. We evaluated the effect of combined treatment of bortezomib with PS on GCT cell lines in vitro but this combination barely showed an additive cytoreductive effect, probably because both cell lines were much susceptible to bortezomib, as it was reported that bortezomib alone strongly inhibited cell growth of these cells at low dose [35].

Next, we examined the effect of combined treatment of PS with a deubiqutinase inhibitor, VLX1570 on KGN and COV434 in vitro. VLX1570 is 19S proteasome inhibitor and inhibits the removal of ubiquitin chains from ubiqutinated proteins by UCHL5 and USP14, distinct from 20S proteasome inhibitor, bortezomib. Hillert EK, et al. examined the effect of combination of a similar deubiqutinase inhibitor, b-AP15 with PS on HCT116 and Hela cell lines and found that the combined treatment of PS with b-AP15 enhanced the proteotoxicity but it did not augment an anti-proliferative effect in those two cell lines [36]. In our study, the combined treatment of PS with VLX1570 additively suppressed the proliferation of KGN and COV434 and induced apoptosis (Fig 2a and 2b). To identify the reason why the combined treatment of PS with VLX1570 showed the additive effect, we examined proteins related to cell cycle progression, apoptosis, the generation of ROS and the histone acetylation by immunoblotting analysis and demonstrated the enhancement of the expression of p21, cPARP, caspase-9, heme oxygenase-1 (HO-1), and the acetylation of histone H4 and α-tubulin in KGN and COV434 (Fig 2c). Hui KF, et al. reported that enhanced histone acetylation was observed after treatment with bortezomib/ class I HDAC inhibitor, romidepsin and could also be reduced upon coincubation of the drug combination with either pan-caspase inhibitor, Z-VAD-FMK or ROS scavenger, N-acetylcysteine (NAC), suggesting that the enhanced histone acetylation was ROS- and caspase-dependent in nasopharyngeal carcinoma (NPC) cells [37, 38]. In our experiment, enhanced expression of HO-1, caspase-9 and histone acetylation may contribute the additive effect by the combined treatment of PS with VLX1570 (Fig 2c).

To further know the action mechanisms of PS and the gene targets of PS, we examined gene expression profiling of PS-treated KGN cells and performed GSEA. From GSEA using the data sets of KEGG pathways, the ontology groups “DNA replication”, “cell cycle”, “spliceosome”, “homologous recombination”, “mismatch repair” and “pyrimidine metabolism” were listed as markedly down-regulated gene sets with PS treatment, whereas the groups “adherens junction”, “endocytosis”, “cell adhesion molecules” and “axon guidance” were listed as significantly up-regulated gene sets with PS treatment. Most of down-regulated KEGG pathway gene sets with PS treatment could be explained as a result of the treatment with an anti-cancer agent. In contrast, among the groups listed as significantly up-regulated gene sets with PS treatment, we focused on “adherens junction”. Kranc et al. reported that genes responsible for proliferation, differentiation, and junction adhesion are up-regulated in human ovarian granulosa cells during in vitro culture and firstly raised the up-regulation of “adherens junction” in KEGG pathways [39]. Their finding is based on the changes of gene expression profiling of cultured normal granulosa cells in vitro, but our study might observe a part of restored gene expression of the tumor cell line as a result of PS treatment.

From GSEA using the data sets of reactome pathways, the ontology groups “mitotic spindle checkpoint”, “cell cycle checkpoints”, “resolution of sister chromatid cohesion”, “cell cycle” and “homology directed repair through homologous recombination” were listed as markedly down-regulated gene sets with PS treatment, whereas the groups “neurotransmitter release cycle”, “serotonin neurotransmitter release cycle”, “L1CAM interactions”, “dopamine neurotransmitter release cycle” and “acetylcholine neurotransmitter release cycle” were listed as markedly up-regulated gene sets with PS treatment.

As for down-regulated gene sets with PS treatment, the cytoreductive effects of PS were shown almost in line with the data from KEGG pathways. As for up-regulated gene sets with PS treatment, surprisingly, neurotransmitter- or neuron-related gene sets were accumulated. These data appeared intriguing but several previous reports suggested the possible relation of granulosa cells and neuronal subjects. Kossowska-Tomaszczuk et al. indicated that granulosa cells can acquire features of neuron under differentiating factors [40]. Up-regulation of NCS1 gene may play a role in the differentiation of granulosa cell towards neuronal cells [36]. Bence et al. reported that a HDAC inhibitor trichostatin A induced up-regulation of monoaminergic neurotransmission genes in neuroblastoma cells [41]. A similar gene-activation effect might occur ectopically on granulosa cell tumor cells, or perhaps it would imply some aspects of neuronal differentiation of granulosa cells. An axonal guidance factor or related gene sets were reported to be involved in granulosa cell function [42, 43], and the axon guidance was actually one of up-regulated gene sets with PS treatment in KEGG pathways, as above described. Taken together, GSEA revealed that PS treatment suppressed DNA replication- or cell cycle-related gene expression which led to chemotherapeutic cell death and in addition, induced activation of adherens junction towards a normalized direction as well as activation of neuron-related gene sets that might imply unexpected differentiation potential due to epigenetic modification by a HDAC inhibitor.

Next, we confirmed that PS increased the expression of E-cadherin (gene symbol: *CDH1*) associated with adherens junction by quantitative RT-PCR and immunoblot analysis (Fig 3b and 3c). In general, E-cadherin is localized at the cell membrane, plays a critical role in epithelial cell-cell adhesion and mediates through the cadherin-catenin adhesion complex. The reduction of E-cadherin expression causes the acquisition of invasive and metastatic phenotype [44, 45]. On the other hand, most of adult granulosa cell tumors showed E-cadherin nuclear expression. Although the role of E-cadherin is not clear in granulosa cells, it may be important as an activator of adherens junction towards a normalized direction in granulosa cell tumors.

In the present study, we investigated the effect of a HDAC inhibitor, PS by using GCT and ovarian cancer cell lines and demonstrated that PS exerted anti-proliferative effect mainly by apoptosis and also possess another potency such as activation of adherens junction toward a normalized direction. Further study regarding potential availability of PS will be needed to establish a promising therapeutic strategy for the patients with GCTs.

## Supporting information

S1 Fig Cell counting by trypan blue staining. Exposure of normal CD34 positive progenitor cells to 10–100 nM of panobinostat (PS) for 0–96 hr decreased the number of living cells. Solid line: normal CD34+ progenitor cells treated with DMSO. Dotted line: normal CD34+ progenitor cells treated with 10–100 nM of PS.
(PDF)

S2 Fig The original images of the immunoblotting analysis in Fig 1b. The same samples were loaded on four gels. The areas shown in Fig 1b were indicated by red frames of the original gels. Ac-H4: acetyl-H4 (Lys12), Ac-tubulin: acetyl-α-tubulin (Lys40), cPARP: cleaved PARP.
(PDF)

S3 Fig The original images of the immunoblotting analysis in Fig 1e. The same samples were loaded on two gels. The areas shown in Fig 1e were indicated by red frames of the original gels.
(PDF)

S4 Fig The original images of the immunoblotting analysis in Fig 2c. The same samples were loaded on two gels. The areas shown in Fig 2c were indicated by red frames of the original gels. cPARP: cleaved PARP, HO-1: heme-oxygenase-1, c-caspase 9: cleaved caspase 9, Ac-H4: acetyl-H4 (Lys12), Ac-tubulin: acetyl-α-tubulin (Lys40).
(PDF)

S5 Fig The heat map presentation of affected genes included in the gene sets. The heat map presentation of affected genes included in the gene sets is shown as each of the triplicate experiments (PS: PS-treated, DMSO: untreated).
(PDF)

S6 Fig Expression of *E-Cadherin* mRNA per an equal amount of total RNA in Fig 3c. Expression of *E-cadherin* mRNA (*CDH1*) per an equal amount of total RNA increased after the treatment with 100–200 nM of PS in RT-qPCR.
(PDF)

S7 Fig Expression of *E-cadherin* mRNA normalized by GAPDH. The expression of *E-cadherin* (*CDH1*) mRNA normalized by *GAPDH* also increased after the treatment with 100–200 nM of PS in RT-qPCR.
(PDF)

S8 Fig The original images of the immunoblotting analysis in Fig 3c. The same samples were loaded on two gels. The areas shown in Fig 3c were indicated by red frames of the original gels.
(PDF)

S9 Fig The heat map presentation of affected genes included in the gene sets. The heat map presentation of affected genes included in the gene sets is shown as each of the triplicate experiments (PS: PS-treated, DMSO: untreated).
(PDF)
