# Supplementary material for: Histone deacetylase inhibitor, panobinostat, exerts anti-proliferative effect with partial normalization from aberrant epigenetic states on granulosa cell tumor cell lines
Source: PLoS One. 2022 Jul 8;17(7):e0271245. doi: 10.1371/journal.pone.0271245 (PMC9269920; doi:10.1371/journal.pone.0271245)
Supplement: S1 Fig — Exposure of normal CD34 positive progenitor cells to 10–100 nM of panobinostat (PS) for 0–96 hr decreased the number of living cells. Solid line: normal CD34+ progenitor cells treated with DMSO. Dotted line: normal CD34+ progenitor cells treated with 10–100 nM of PS. (PDF) [file pone.0271245.s001.pdf]

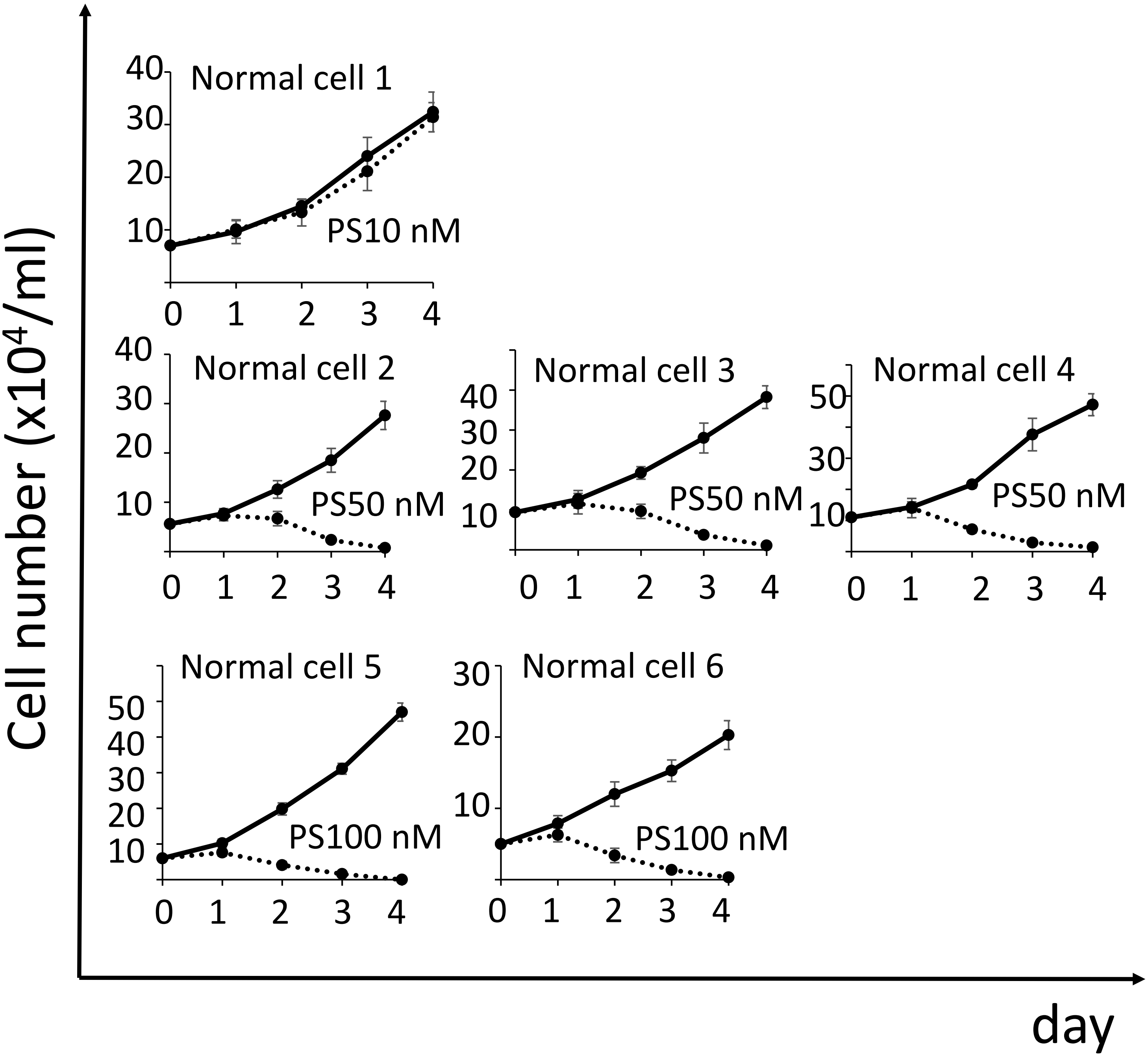

S1 Fig. Cell counting by trypan blue staining

Exposure of normal CD34 positive progenitor cells to 10-100 nM of panobinostat (PS) for 0-96 hr decreased the number of living cells.

Solid line: normal CD34+ progenitor cells treated with DMSO

Dotted line: normal CD34+ progenitor cells treated with 10-100 nM of PS
