# Supplementary material for: Histone deacetylase inhibitor, panobinostat, exerts anti-proliferative effect with partial normalization from aberrant epigenetic states on granulosa cell tumor cell lines
Source: PLoS One. 2022 Jul 8;17(7):e0271245. doi: 10.1371/journal.pone.0271245 (PMC9269920; doi:10.1371/journal.pone.0271245)
Supplement: S2 Fig — The same samples were loaded on four gels. The areas shown in Fig 1b were indicated by red frames of the original gels. Ac-H4: acetyl-H4 (Lys12), Ac-tubulin: acetyl-α-tubulin (Lys40), cPARP: cleaved PARP. (PDF) [file pone.0271245.s002.pdf]

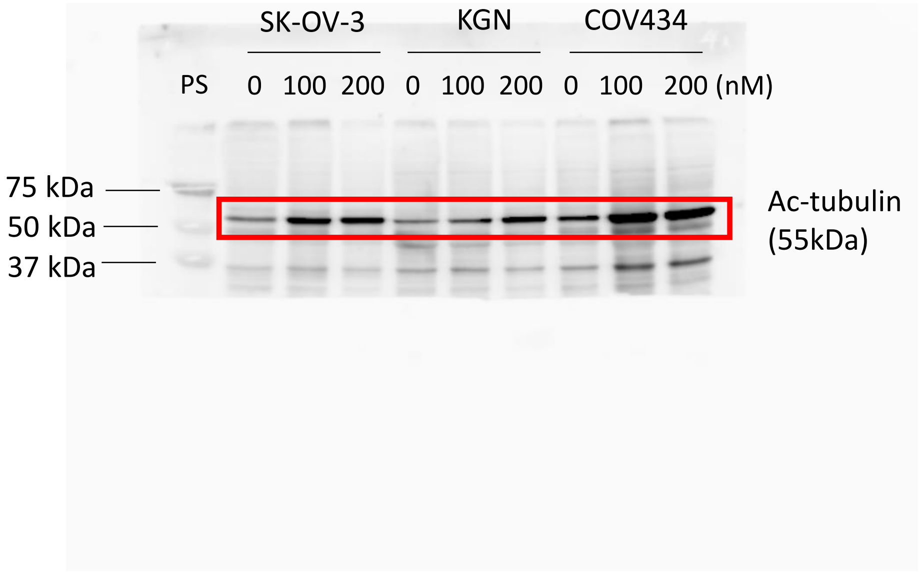
SK-OV-3
KGN
COV434
PS
0 100 200 0 100 200 0 100 200 (nM)
75 kDa
50 kDa
37 kDa
Ac-tubulin
(55kDa)

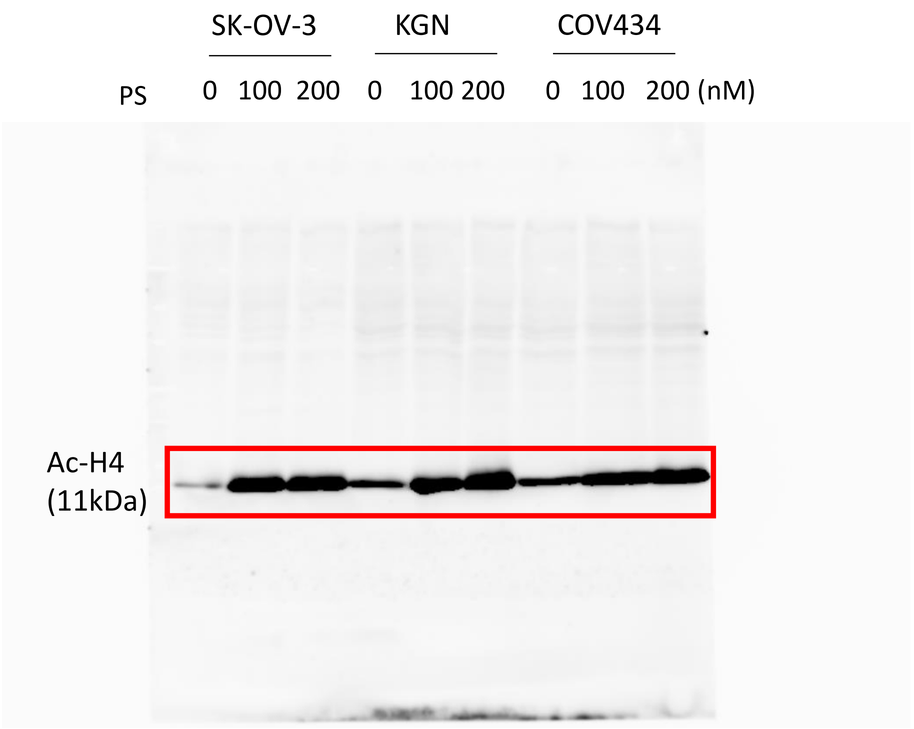
SK-OV-3
KGN
COV434
PS
0 100 200 0 100 200 0 100 200 (nM)
Ac-H4
(11kDa)

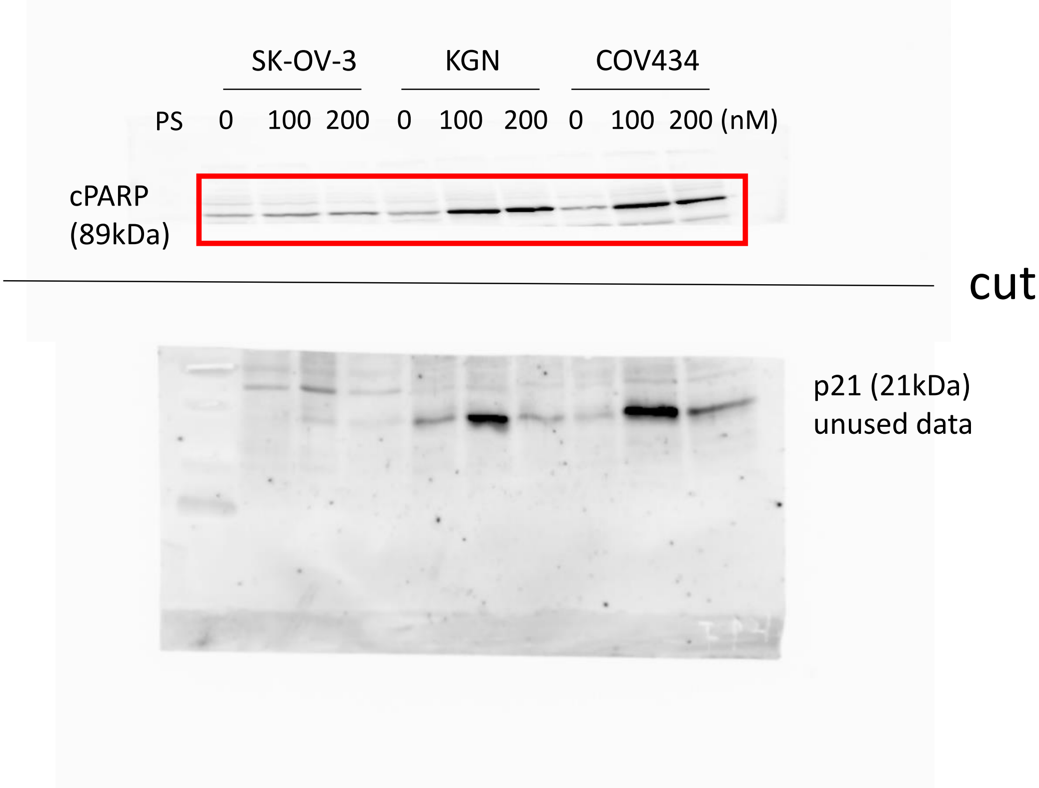
SK-OV-3
KGN
COV434
PS 0 100 200 0 100 200 0 100 200 (nM)
cPARP
(89kDa)
cut
p21 (21kDa)
unused data

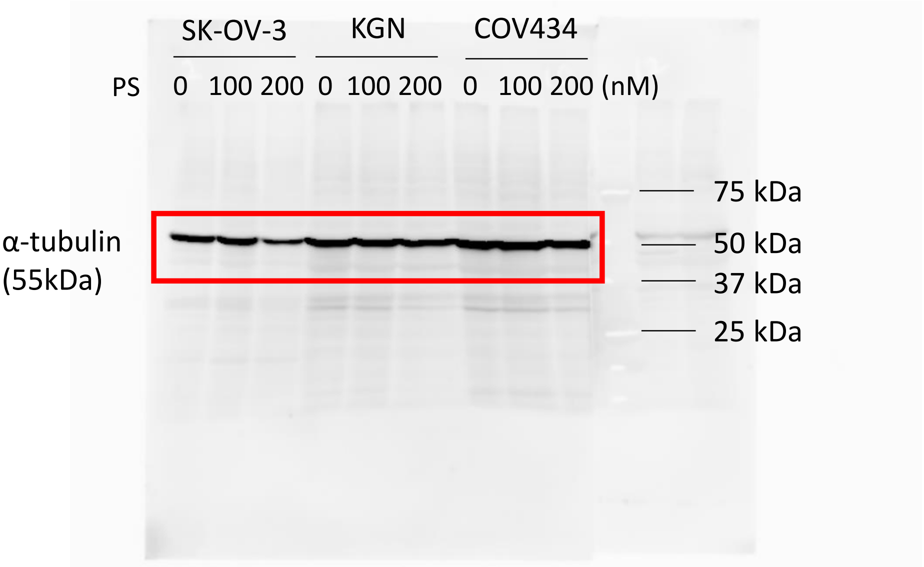
SK-OV-3
KGN
COV434
PS 0 100 200 0 100 200 0 100 200 (nM)
α-tubulin
(55kDa)
75 kDa
50 kDa
37 kDa
25 kDa

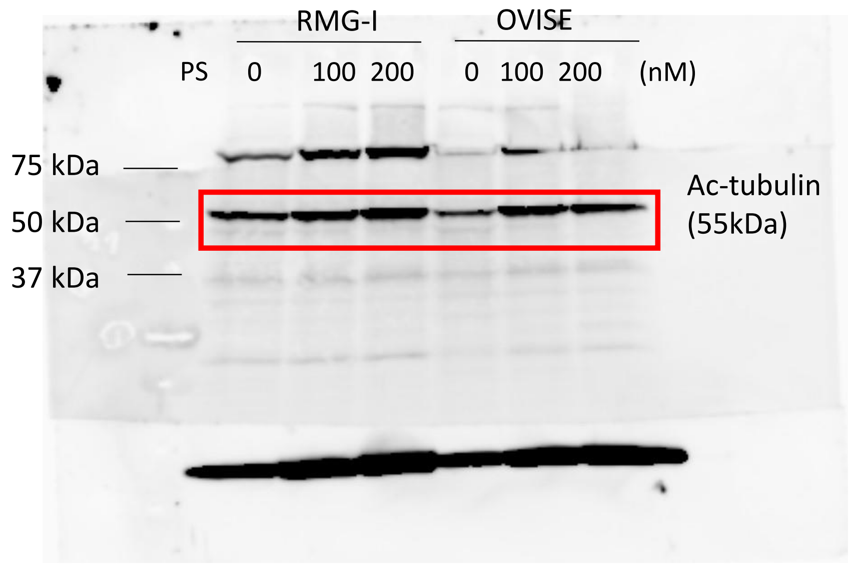
RMG-I
OVISE
PS
0
100
200
0
100
200
(nM)
75 kDa
50 kDa
37 kDa
Ac-tubulin
(55kDa)

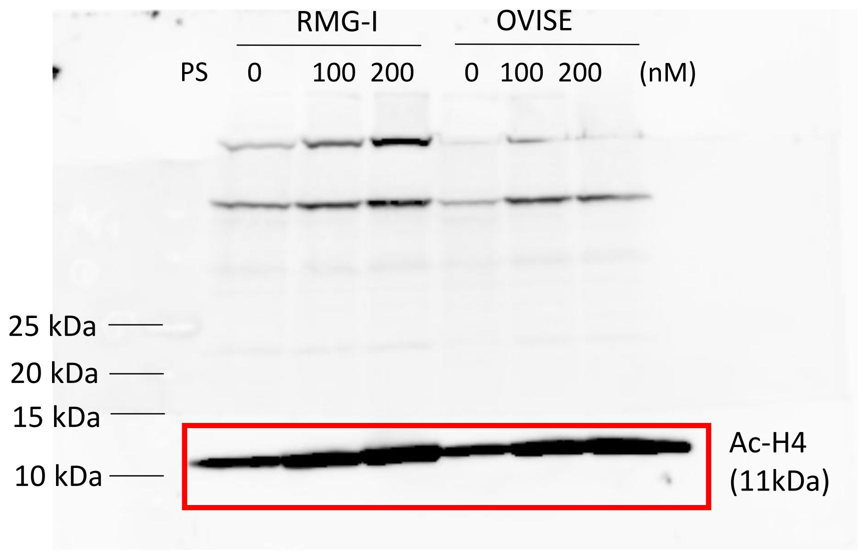
RMG-I
OVISE
PS
0
100
200
0
100
200
(nM)
25 kDa
20 kDa
15 kDa
10 kDa
Ac-H4
(11kDa)

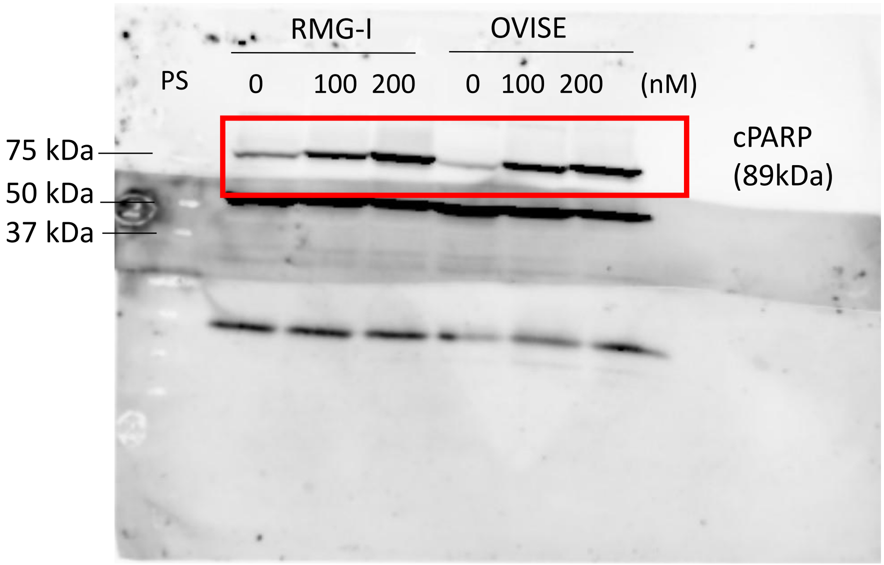

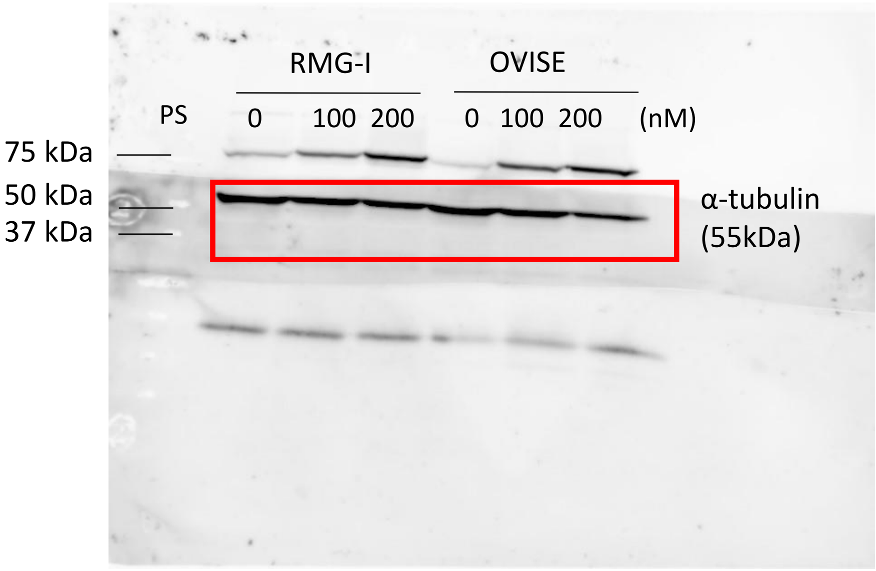

S2 Fig. The original images of the immunoblotting analysis in Fig 1b

The same samples were loaded on four gels. The areas shown in Fig 1b were indicated by red frames of the original gels.

Ac-H4: acetyl-H4 (Lys12), Ac-tubulin: acetyl-α-tubulin (Lys40), cPARP: cleaved PARP
