# Supplementary material for: Histone deacetylase inhibitor, panobinostat, exerts anti-proliferative effect with partial normalization from aberrant epigenetic states on granulosa cell tumor cell lines
Source: PLoS One. 2022 Jul 8;17(7):e0271245. doi: 10.1371/journal.pone.0271245 (PMC9269920; doi:10.1371/journal.pone.0271245)
Supplement: S3 Fig — The same samples were loaded on two gels. The areas shown in Fig 1e were indicated by red frames of the original gels. (PDF) [file pone.0271245.s003.pdf]

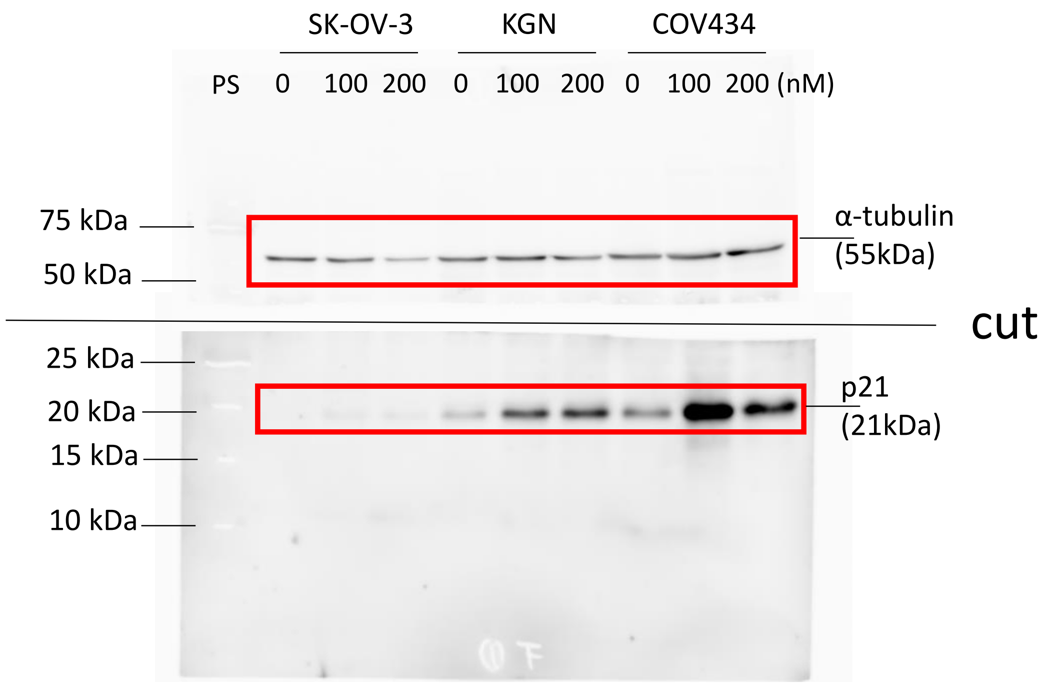
SK-OV-3
KGN
COV434
PS
0
100
200
0
100
200
0
100
200 (nM)
75 kDa
50 kDa
α-tubulin
(55kDa)
cut
25 kDa
20 kDa
15 kDa
10 kDa
p21
(21kDa)

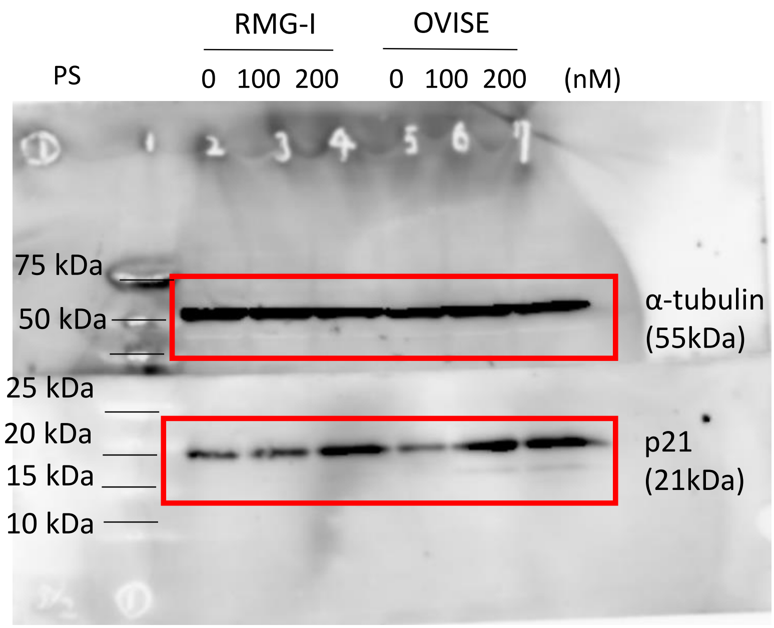

S3 Fig. The original images of the immunoblotting analysis in Fig 1e

The same samples were loaded on two gels. The areas shown in Fig 1e were indicated by red frames of the original gels.
