# Supplementary material for: Histone deacetylase inhibitor, panobinostat, exerts anti-proliferative effect with partial normalization from aberrant epigenetic states on granulosa cell tumor cell lines
Source: PLoS One. 2022 Jul 8;17(7):e0271245. doi: 10.1371/journal.pone.0271245 (PMC9269920; doi:10.1371/journal.pone.0271245)
Supplement: S4 Fig — The same samples were loaded on two gels. The areas shown in Fig 2c were indicated by red frames of the original gels. cPARP: cleaved PARP, HO-1: heme-oxygenase-1, c-caspase 9: cleaved caspase 9, Ac-H4: acetyl-H4 (Lys12), Ac-tubulin: acetyl-α-tubulin (Lys40). (PDF) [file pone.0271245.s004.pdf]

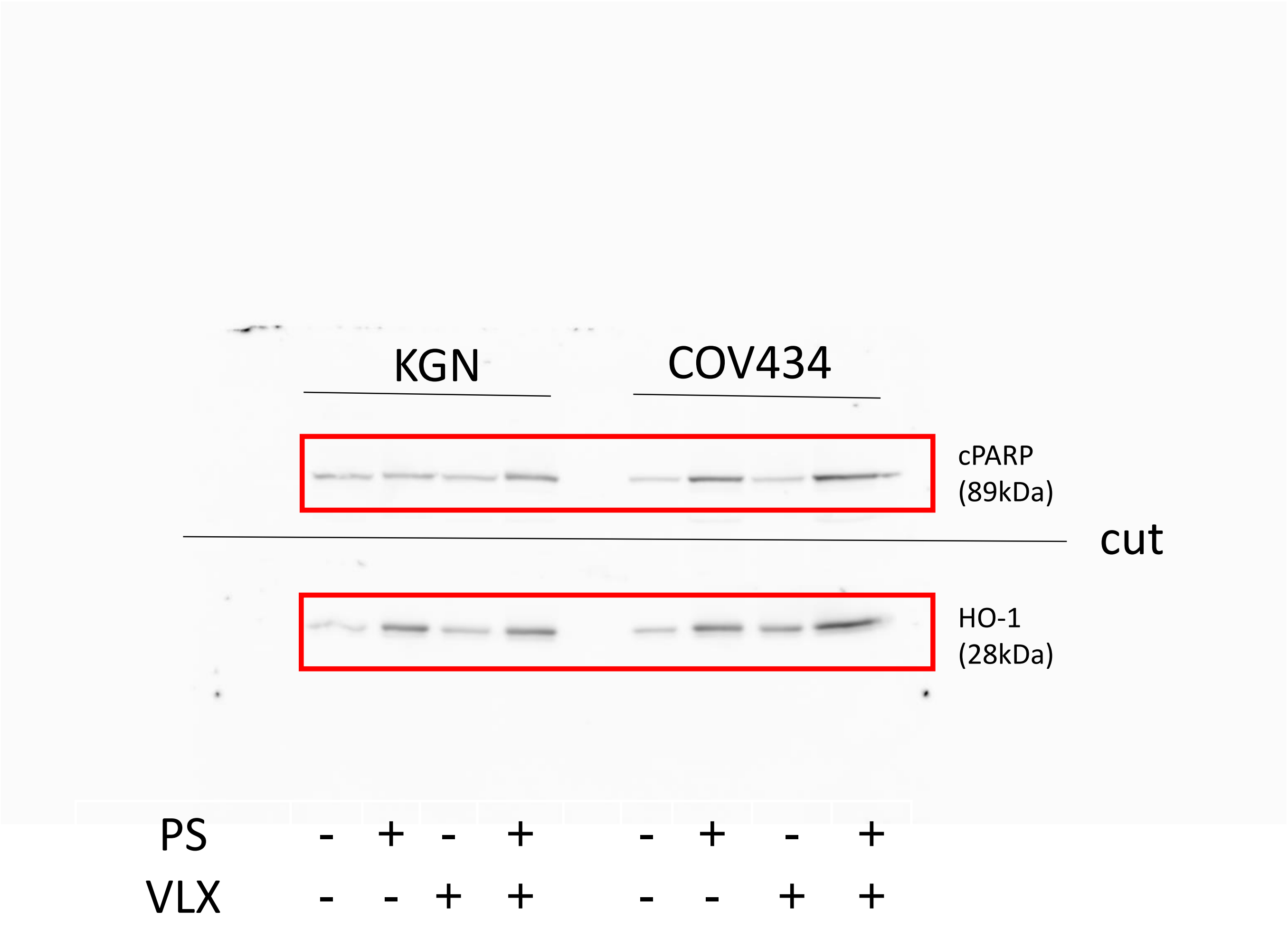
KGN
COV434
cPARP
(89kDa)
cut
HO-1
(28kDa)
PS - + - + - + - +
VLX - - + + - - + +

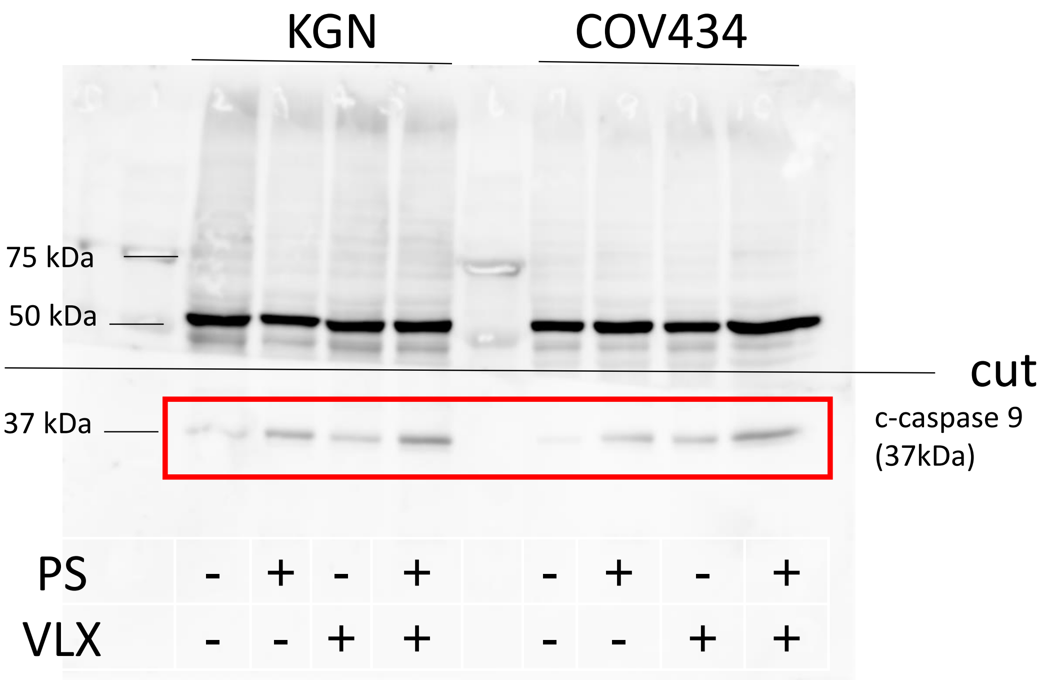
KGN
COV434
75 kDa
50 kDa
cut
37 kDa
c-caspase 9
(37kDa)
PS - + - + - + - +
VLX - - + + - - + +

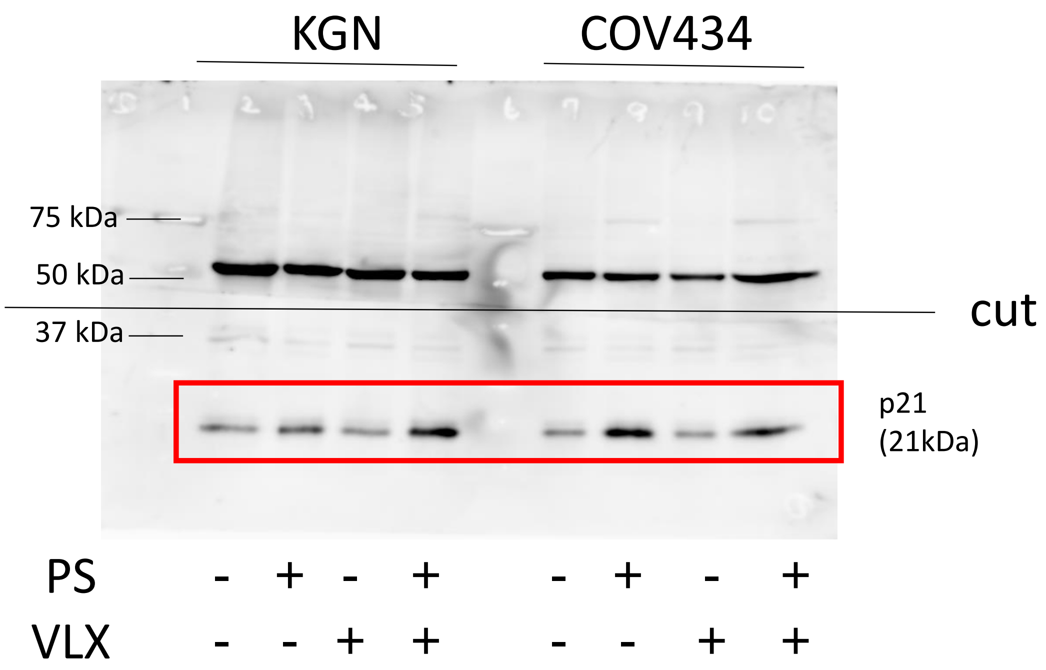
KGN
COV434
75 kDa
50 kDa
37 kDa
cut
p21
(21kDa)
PS - + - + - + - +
VLX - - + + - - + +

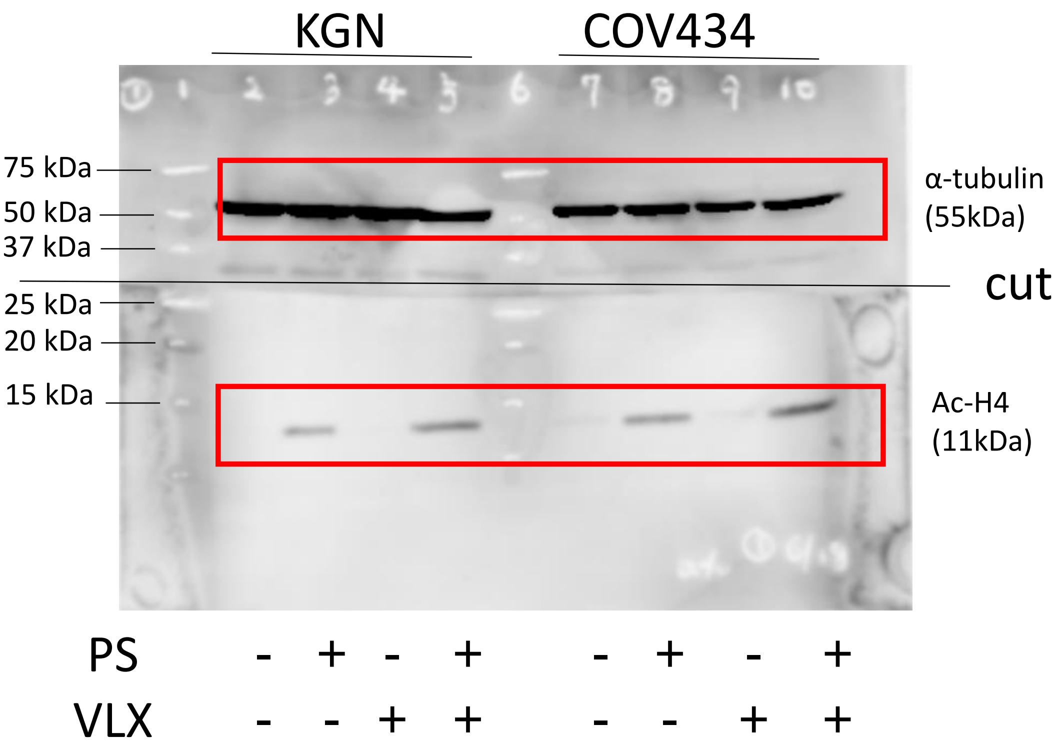
KGN
COV434
75 kDa
50 kDa
37 kDa
25 kDa
20 kDa
15 kDa
α-tubulin
(55kDa)
cut
Ac-H4
(11kDa)
PS - + - + - + - +
VLX - - + + - - + +

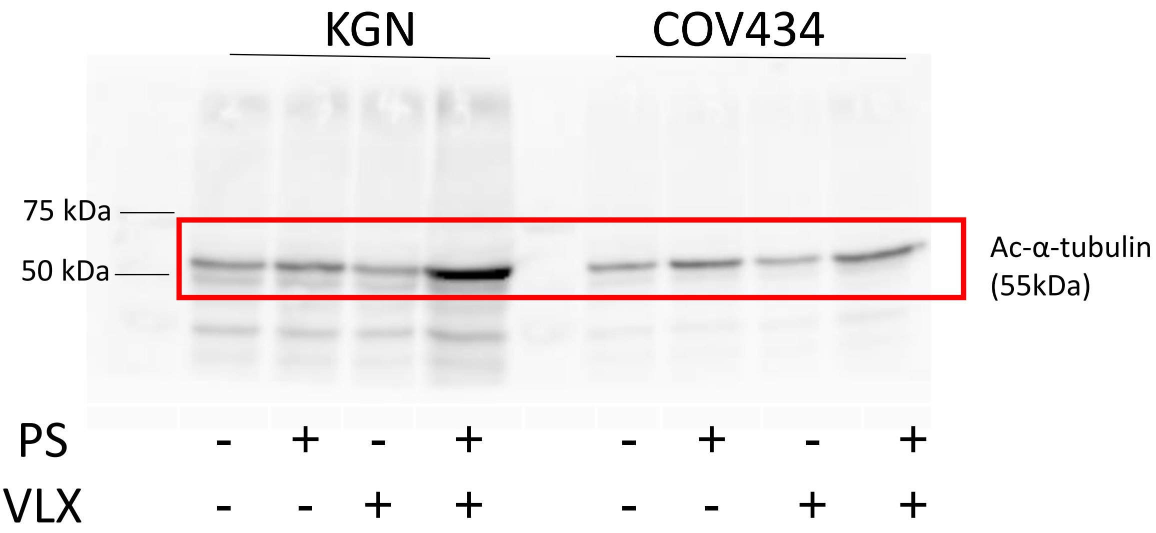

S4 Fig. The original images of the immunoblotting analysis in Fig 2c

The same samples were loaded on two gels. The areas shown in Fig 2c were indicated by red frames of the original gels.

cPARP: cleaved PARP, HO-1: heme-oxygenase-1, c-caspase 9: cleaved caspase 9, Ac-H4: acetyl-H4 (Lys12), Ac-tubulin: acetyl-α-tubulin (Lys40)
