# Supplementary figures and images for: Histone deacetylase inhibitor, panobinostat, exerts anti-proliferative effect with partial normalization from aberrant epigenetic states on granulosa cell tumor cell lines
Source: PLoS One. 2022 Jul 8;17(7):e0271245. doi: 10.1371/journal.pone.0271245 (PMC9269920; doi:10.1371/journal.pone.0271245)

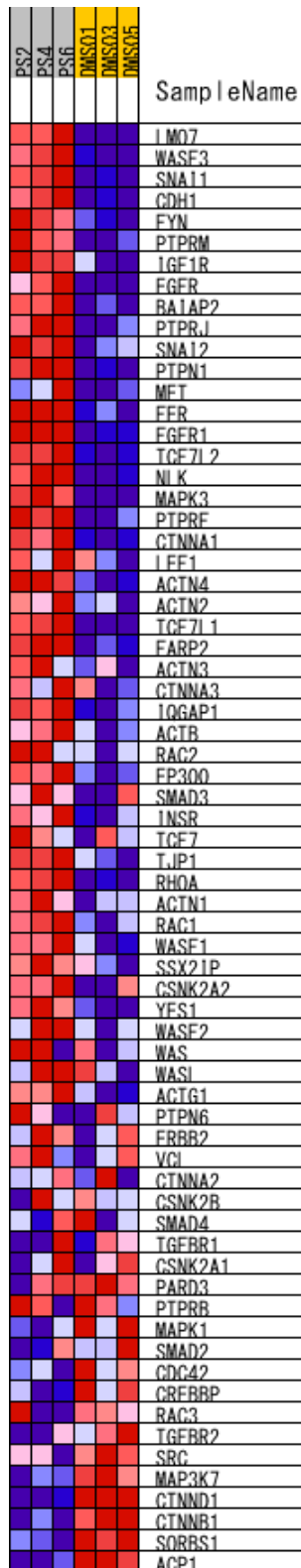

adherens junction

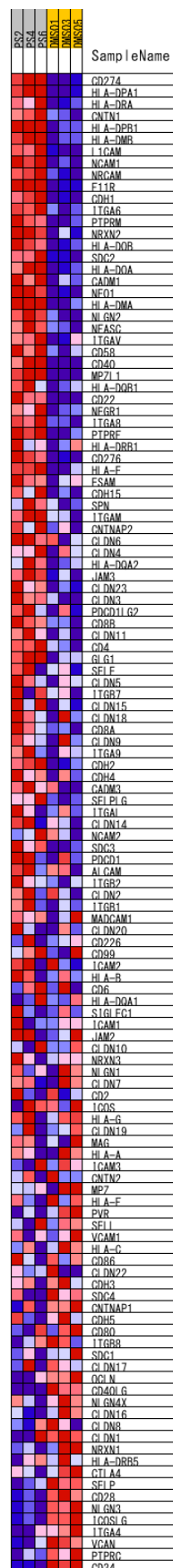

cell adhesion  
molecules

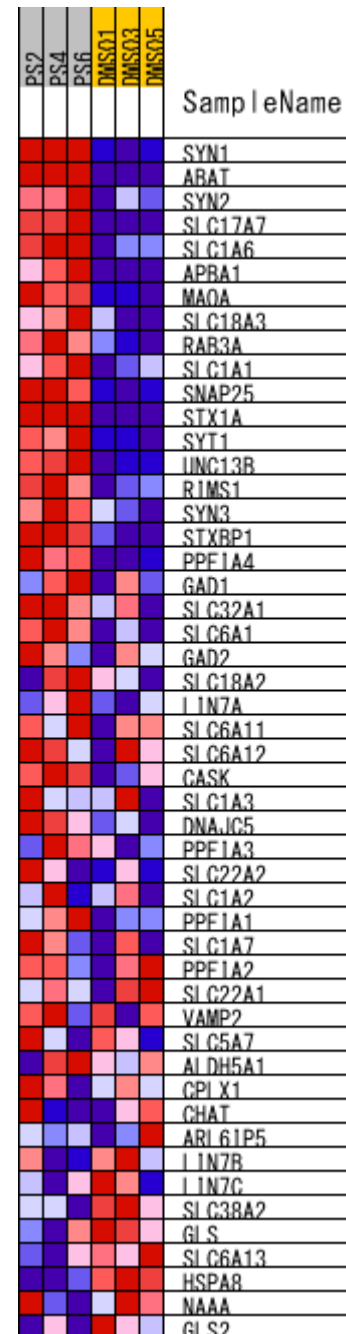

Supplement: S5 Fig — The heat map presentation of affected genes included in the gene sets is shown as each of the triplicate experiments (PS: PS-treated, DMSO: untreated). (PDF) [file pone.0271245.s005.pdf]
