# Supplementary material for: Histone deacetylase inhibitor, panobinostat, exerts anti-proliferative effect with partial normalization from aberrant epigenetic states on granulosa cell tumor cell lines
Source: PLoS One. 2022 Jul 8;17(7):e0271245. doi: 10.1371/journal.pone.0271245 (PMC9269920; doi:10.1371/journal.pone.0271245)
Supplement: S6 Fig — Expression of E-cadherin mRNA (CDH1) per an equal amount of total RNA increased after the treatment with 100–200 nM of PS in RT-qPCR. (PDF) [file pone.0271245.s006.pdf]

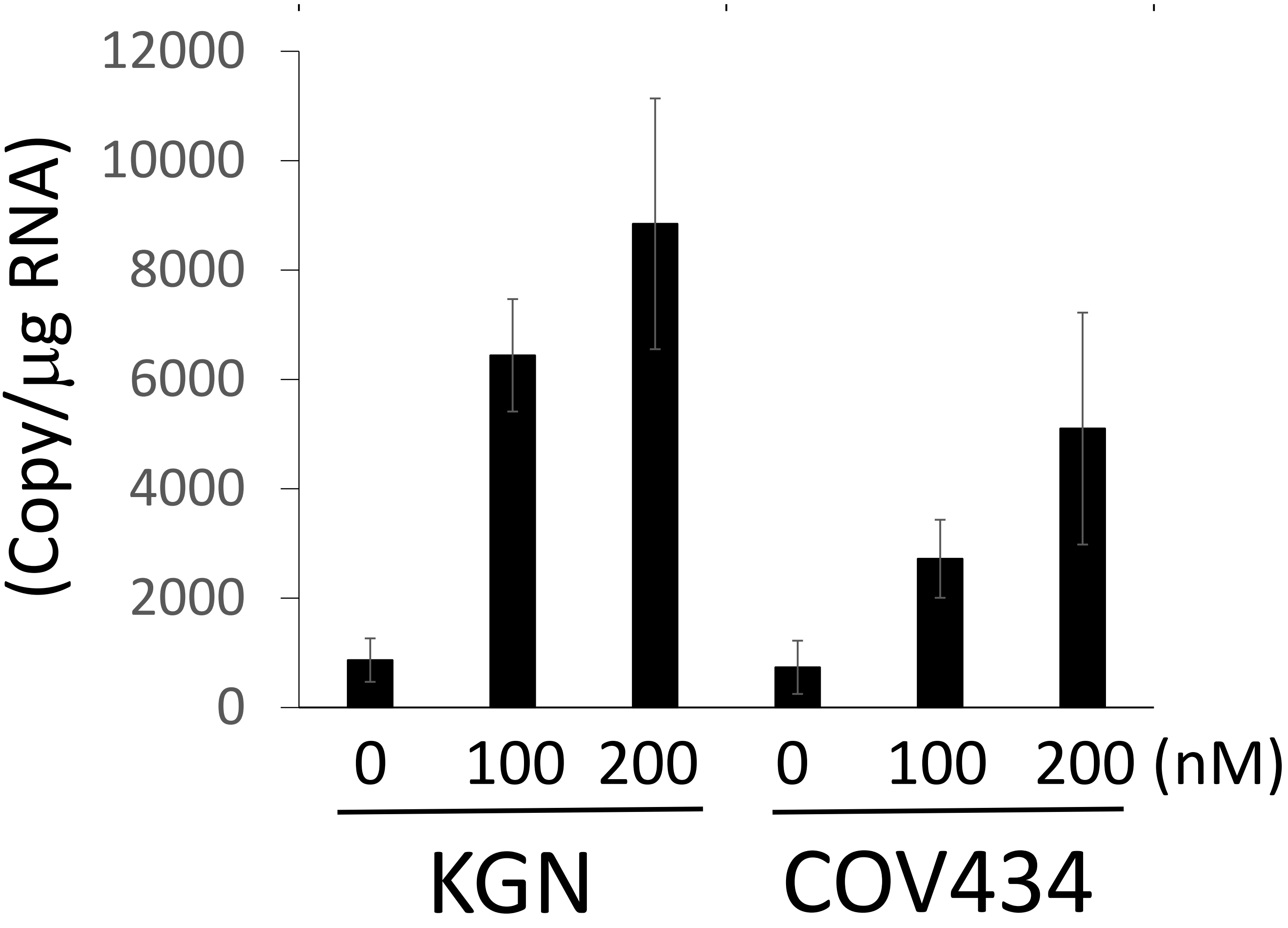

S6 Fig. Expression of *E-Cadherin* mRNA per an equal amount of total RNA in Fig 3c

Expression of *E-cadherin* (*CDH1*) mRNA per an equal amount of total RNA increased after the treatment with 100-200 nM of PS in RT-qPCR.
