# Supplementary material for: Histone deacetylase inhibitor, panobinostat, exerts anti-proliferative effect with partial normalization from aberrant epigenetic states on granulosa cell tumor cell lines
Source: PLoS One. 2022 Jul 8;17(7):e0271245. doi: 10.1371/journal.pone.0271245 (PMC9269920; doi:10.1371/journal.pone.0271245)
Supplement: S8 Fig — The same samples were loaded on two gels. The areas shown in Fig 3c were indicated by red frames of the original gels. (PDF) [file pone.0271245.s008.pdf]

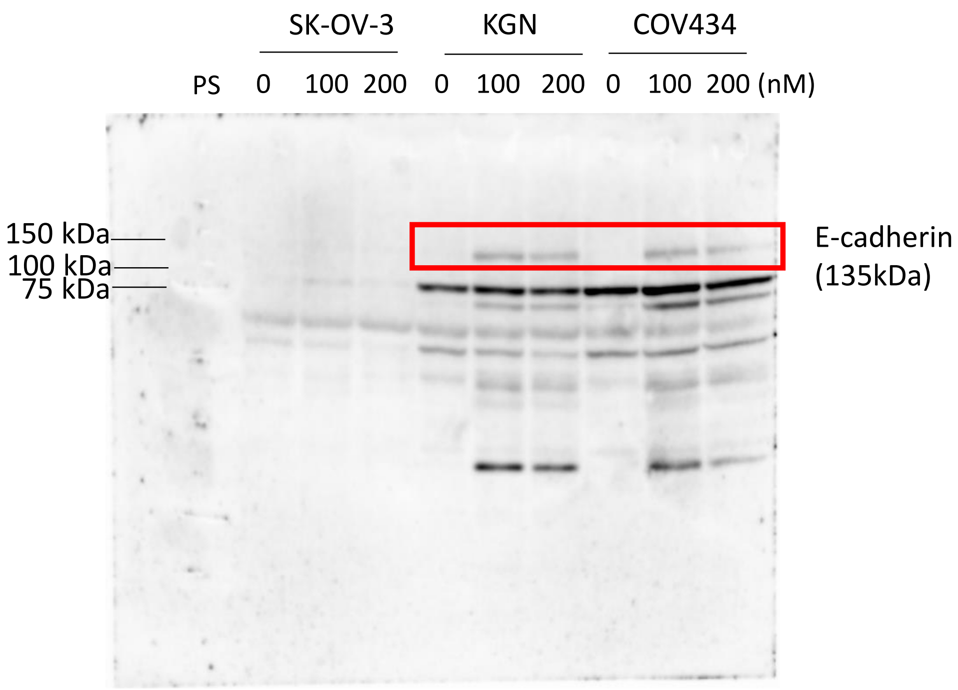

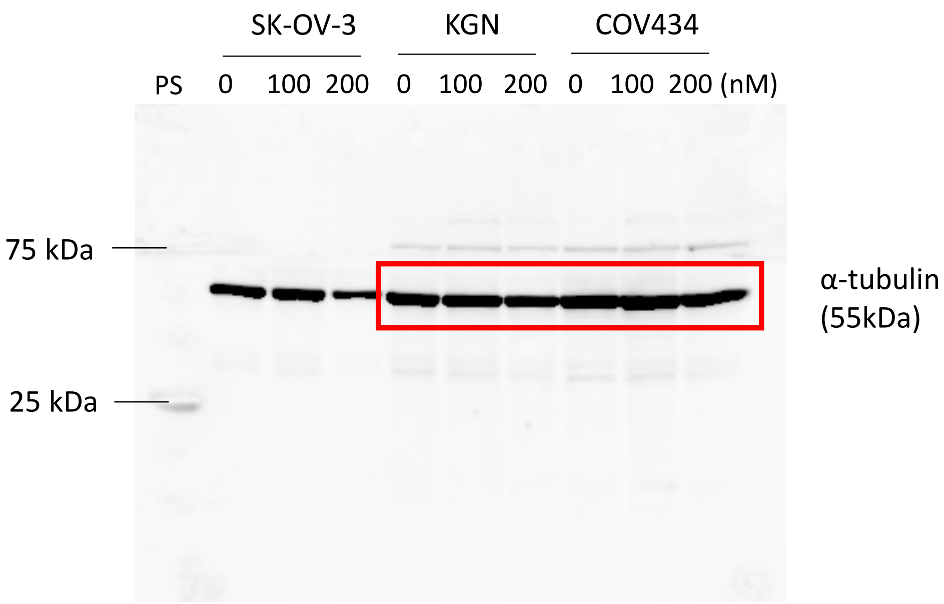

S8 Fig. The original images of the immunoblotting analysis in Fig 3c

The same samples were loaded on two gels. The areas shown in Fig 3c were indicated by red frames of the original gels.
