# Supplementary material for: Histone deacetylase inhibitor, panobinostat, exerts anti-proliferative effect with partial normalization from aberrant epigenetic states on granulosa cell tumor cell lines
Source: PLoS One. 2022 Jul 8;17(7):e0271245. doi: 10.1371/journal.pone.0271245 (PMC9269920; doi:10.1371/journal.pone.0271245)
Supplement: S9 Fig — The heat map presentation of affected genes included in the gene sets is shown as each of the triplicate experiments (PS: PS-treated, DMSO: untreated). (PDF) [file pone.0271245.s009.pdf]

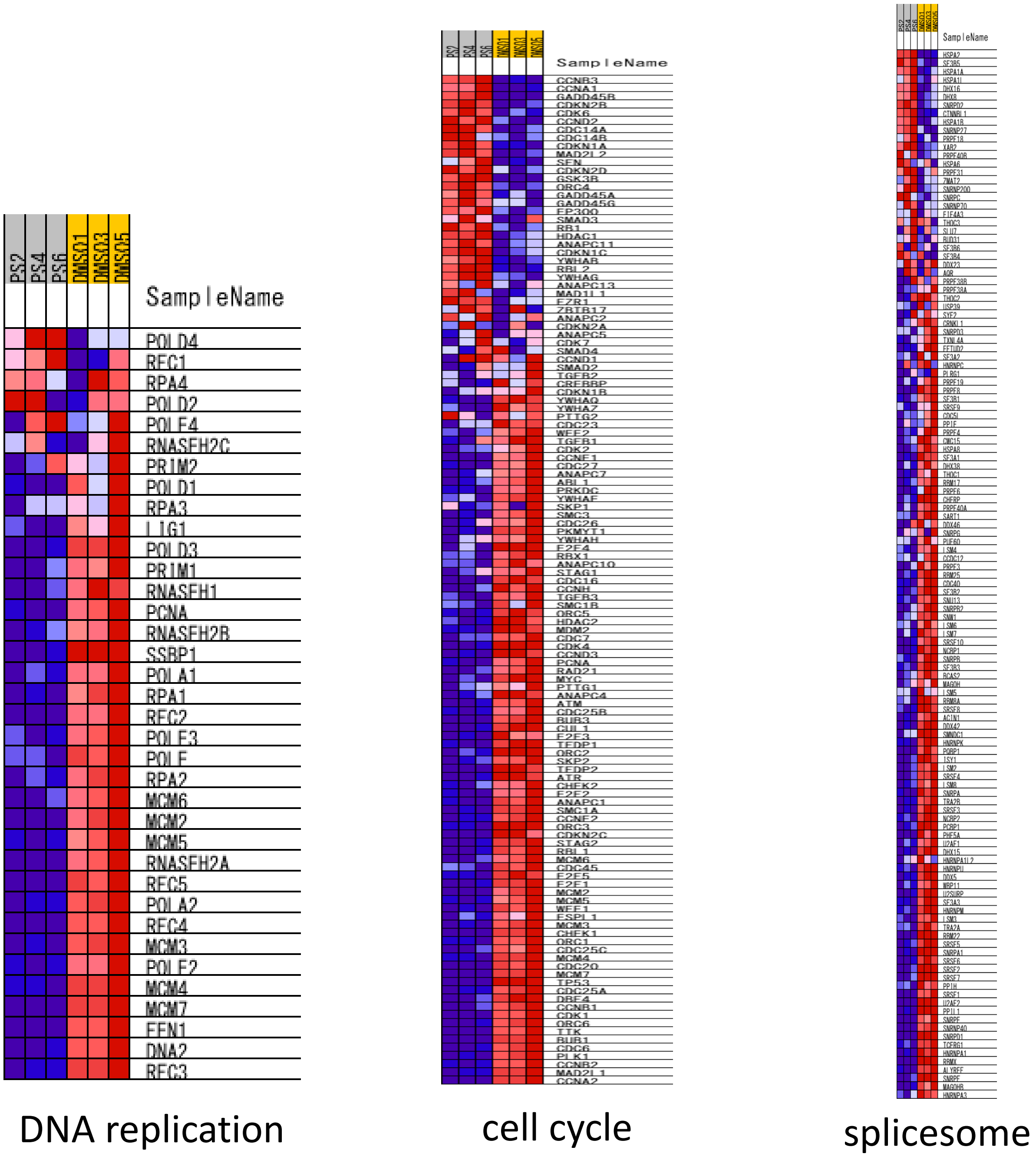

S9 Fig. The heat map presentation of affected genes included in the gene sets

The heat map presentation of affected genes included in the gene sets is shown as each of the triplicate experiments (PS: PS-treated, DMSO: untreated).
